# The Interaction of CD97/ADGRE5 With β-Catenin in Adherens Junctions Is Lost During Colorectal Carcinogenesis

**DOI:** 10.3389/fonc.2018.00182

**Published:** 2018-05-25

**Authors:** Doris Hilbig, Norman Dietrich, Elke Wandel, Susann Gonsior, Doreen Sittig, Jörg Hamann, Gabriela Aust

**Affiliations:** ^1^Department of Surgery, Research Laboratories, Leipzig University, Leipzig, Germany; ^2^Department of Experimental Immunology, Academic Medical Centre, University of Amsterdam, Amsterdam, Netherlands

**Keywords:** ADGRE5, CD97, adhesion-GPCR, β-catenin, adherens junction, colorectal carcinoma

## Abstract

The adhesion G-protein-coupled receptor CD97/ADGRE5 is present in adherens junctions of human normal intestinal cells and upregulated in colorectal carcinomas. Here, we examined whether CD97 directly interacts with junctional proteins in normal and malignant colorectal tissue. We identified an association of CD97 with β-catenin using a proximity ligation assay and confirmed the interaction between both endogenous proteins at the biochemical level by co-immunoprecipitation in human and mouse tissues and cell lines. Glutathione S-transferase-pulldown revealed that CD97 binds β-catenin through its seven-span transmembrane/intracellular domain(s). To study tumor-associated changes in the interaction of CD97 and β-catenin *in situ*, we quantified and correlated both proteins at the membrane, and in the cytoplasm and nuclei of colorectal carcinomas and their corresponding normal tissues (*n* = 111). In normal colon, membranous levels of CD97 and β-catenin correlated strongly (*p* < 0.0001). To some degree both molecules disappeared in carcinomas simultaneously from the membrane of tumor cells (*p* = 0.017). CD97 accumulated in the cytoplasm, whereas β-catenin emerged in the cytoplasm and nuclei. CD97 and β-catenin levels in the cytoplasm correlated well (*p* < 0.0001). Irrespective of their subcellular localization, interaction of CD97 with β-catenin in tumor cells was also restricted to the cell contacts. Accordingly, CD97 did not regulate β-catenin-dependent TCF-mediated transcriptional activity. In summary, while CD97 and β-catenin interact in adherens junctions, their interaction is lost and both molecules follow different functional paths inside tumor cells.

## Introduction

Epithelial cells stick together by lateral cell contacts in which molecules, bridging the intercellular space, are anchored by connecting proteins to the cytoskeleton, ensuring tightening, mechanical stability, paracellular transport, and signal transduction (1). During carcinogenesis, epithelial junctions are remodeled and/or disintegrated, and junctional proteins disappear from the cell contacts and translocate into or accumulate in the cytoplasm and/or nuclei (2–4). Their relocation implies changes in the function of junctional proteins, caused by new molecular interactions. This was demonstrated convincingly for β-catenin. In adherens junctions it links the transmembrane receptor E-cadherin (Ecad), which mediates strong homotypic adhesion between neighboring cells, *via* connecting proteins to the actin cytoskeleton (5). In contrast, accumulated in the nucleus, β-catenin binds to TCF/LEF transcription factors, activating genes driving colorectal carcinogenesis (6).

CD97/ADGRE5 is a prototypic member of the adhesion family of G-protein coupled receptors (aGPCRs). The large extracellular domain (ECD) of CD97 with numerous adhesive EGF-like folds and the GPCR autoproteolysis-inducing (GAIN) domain enables adhesion of leukocytes to other surface receptors and extracellular matrix constituents such as CD55 (7), chondroitin sulfate B (8), α5β1 and αvβ3 integrins (9), and CD90 (10). Typical for aGPCRs, autocatalytic cleavage within the GAIN domain results in non-covalently bound CD97 N-terminal (NTF) and C-terminal (CTF) fragments (11). The ECD of aGPCRs passes into the seven-span transmembrane helices (TM7) with the intracellular tail.

Recently, we localized CD97 in adherens junctions of human intestinal epithelial cells (12). While only weakly present in normal enterocytes, CD97 is induced or upregulated in the corresponding carcinomas (13). Especially single or grouped budding tumor cells at the invasive front strongly express CD97, which was related to higher tumor stage and lymphatic vessel infiltration (13). Obviously, the cells showed cytoplasmic CD97. The varying subcellular localization of CD97, which has not been verified systematically, raised the hypothesis that it belongs to those junctional proteins which change their molecular interactions and thus function during tumorigenesis. Beside colorectal carcinomas, CD97 is upregulated and/or biochemically modified in various other malignancies [reviewed in Ref. (14)]. Consistently, CD97 promotes tumor growth and metastatic spread in mouse models of colorectal, gastric, thyroid, and pancreatic cancer, and CD97-silencing regulates migration and invasion of tumor cells *in vitro* (15–18). It mediates prostate and thyroid tumor cell invasion, at least in part, by lysophosphatidic acid (LPA)-dependent coupling to Gα12/13 and RhoA activation (16).

In contrast to malignancies, the knowledge on CD97 function in normal epithelial cells is minimal. In a mesenchymal cell line with heterologous CD97-promoted homotypic cell-cell aggregation *via* upregulation of N-cadherin (19) suggests a CD97-dependent regulation of cell contacts. Consistently, in transgenic mice selectively expressing CD97 in enterocytes, CD97 strengthened normal adherens junctions whereby experimental colitis was CD97 dose-dependently attenuated (20). In these mice CD97 enhanced membrane-bound non-phosphorylated β-catenin (20). The data suggest a CD97-dependent regulation of key junctional proteins such as β-catenin in normal epithelial cells, although their biochemical interaction has not been verified yet. Moreover, the fate of this interaction after malignant transformation is unknown. The present study was initiated to answer these open questions.

Here, using proximity ligation and biochemical assays, we demonstrate that β-catenin is indeed an intracellular interaction partner of CD97 in adherens junctions. During colorectal carcinogenesis, β-catenin emerged in the cytoplasm and nuclei, whereas CD97 accumulated in the cytoplasm of tumor cells. CD97 and β-catenin interaction is almost always restricted to cellular junctions.

## Materials and Methods

### Patients and Mice

The histological study comprised 111 sporadic colorectal adenocarcinomas. Normal mucosal specimens from at least 5-cm away from tumor lesions were obtained in parallel. Histological diagnosis and staging followed the tumor, node, and metastasis classification (21). Hematoxylin–eosin-stained slides were examined for tumor buds, defined as the presence of scattered tumor cells or small tumor cell clusters at the invasive front or within the main tumor body. In colorectal cancer, tumor budding has strong prognostic power (22, 23). Patients were divided into two groups according to the degree of budding: none or mild and moderate or severe (22).

Generation of *Cd97* knock-out (Ko) mice and Tg(villin-CD97) mice, expressing CD97 in intestinal epithelial cells, has been described previously (20, 24).

### Antibodies (Abs)

The following Abs were used: Ecad (sc-7870, Santa Cruz, Heidelberg, Germany), glutathione S-transferase (GST) (MA4-004; Thermo Fisher Scientific, Darmstadt, Germany), α-catenin (GTX22981, GeneTex, Irvine, CA, USA), β-catenin (sc-7199; Santa Cruz; 610153; BD Transduction Laboratories, Heidelberg, Germany), p120-catenin (sc-13957, Santa Cruz), α-tubulin (T9026, Sigma-Aldrich, Munich, Germany), and ZO-1 (61-7300, Thermo Fisher). CD97 was detected using Abs directed to its NTF (H00000976-B01P, Abnova, Taipeh, Taiwan; AF3734, R&D Systems GmbH, Wiesbaden, Germany; 21270971, ImmunoTools, Friesoythe, Germany; HPA013707, Sigma) or CTF (24).

### Immunohistology

5-µm paraffin sections were rehydrated, and endogenous peroxidase activity was blocked with 1.0% hydrogen peroxide/96% ethanol between the steps absolute and 96% ethanol for 5 min. After antigen retrieval (pH 6.0), sections were incubated with primary Abs for 30 min. Ab binding was detected with secondary Abs and the Vectastain Elite ABC kit (Vector Laboratories, Burlingame, CA, USA). Double immunolabelling was performed as described (24).

To correlate the localization of CD97 and β-catenin within the cells, three compartments, i.e., lateral cell contacts, cytoplasm, and nuclei, were examined. The percentage of cells stained within these compartments in normal and tumor colon tissue was ranked between 0 and 4 (0: ≤5, 1: 6–25, 2: 26–50, 3: 51–75, 4: ≥76% positive cells). Staining intensity was estimated between 0 and 3 (0: negative, 1: weak, 2: moderate, 3: strong staining). The product of positive cells and staining intensity gave the score for each compartment (0–12).

### Cell Lines

DLD1, WiDr, Caco-2, SW480 (all colorectal), Saos-2 (osteosarcoma), HT1080 (mesenchymal), and HEK293 (embryonic kidney) cells were obtained from ATCC (LGC Standards GmbH, Wesel, Germany). U-1242MG (glioma), EPLC 272 H (lung), and HT-29/B6 (colorectal) cells were kindly provided by N. E. Heldin (University of Uppsala, Sweden), G. Jaques (University of Marburg, Germany), and M. Fromm (Charité, Berlin, Germany), respectively. The specificity of the cell lines was confirmed by spectral karyotyping and GTG-banding to detect numerical and/or structural chromosomal aberrations. Cells were transfected with lipofectamine 2000 (Thermo Fisher). OmicsLink CD97 shRNA psi-H1 constructs (GeneCopoeia, Rockville, MD, USA) were used to silence CD97 in DLD1 cells. Two CD97 siRNAs were designed: 5′-aaatcaatccagacatgaa-3′ and 5′-cctgcattccaagaagcaa-3′. A scrambled 19-mer shRNA psi-H1 construct was used as control.

Generation of HT1080 clones overexpressing full-length CD97(EGF125), C-terminally truncated CD97(EGF125/TM2), and inversely oriented CD97(EGF125) (mock) has been described (15). In the same way, stable WiDr clones overexpressing a CD97(EGF125)-EGFP fusion protein were generated using the pEGFPN1 plasmid (Clontech, Mountain View, CA, USA). The WiDr clones were treated with 3.2 mM EGTA to disrupt cell contacts. To reform them, the medium was changed to contain 40 mM CaCl_2_ and 20% fetal calf serum. For immunostaining, cultured cells were fixed with ice-cold acetone for 10 min and stained with primary Abs and fluorochrome-labeled secondary Abs as described (24).

### Proximity Ligation Assay (PLA)

To detect protein:protein interaction in cultured cells and tissue sections, the DuoLinkTM *in situ* PLA (Olink Bioscience, Uppsala, Sweden) was used. Paraffin sections were rehydrated, and antigen retrieval (pH 6.0) was performed. Cells cultured on glass coverslips were fixed with ice-cold acetone for 10 min. The slides were incubated with blocking solution for 30 min at 37°C, immunolabelled with primary Abs over night at 4°C, incubated with secondary Abs attached to the PLA probes for 1 h at 37°C, and, finally, incubated with detection solution. Co-immunostaining of one or both antigens was performed after PLA by detecting the primary Abs with suitable fluorochrome-labeled secondary Abs. Nuclei were stained by TO-PRO-3 iodide (Thermo Fisher). The number and localization of interaction spots within the various cellular compartments were quantified with confocal laser scanning microscopy and the image processing program Fiji (http://fiji.sc/).

### GST Pulldown and Co-Immunoprecipitation

Human β-catenin pGEX4T1 (25) was kindly provided by Y. Yang (Genetics Disease Research Branch, NIH, Bethesda, USA). β-catenin-glutathione S-transferase or GST alone were induced by 1 mM isopropyl β-d-1-thiogalactopyranoside (IPTG) in *E. coli* BL21 cells for 4 h at 37°C and purified (25). Proteins were analyzed by SDS-PAGE and Coomassie staining. For pulldown assays using the ProFound™ Pull-Down GST protein:protein interaction kit (Thermo Fisher), GST-tagged protein or GST alone were loaded on columns containing glutathione-agarose beads over night at 4°C. Afterward, the loaded beads were incubated for 16 h at 4°C with lysates of HT1080 clones. Bound proteins were eluted with 100 mM gluthatione and detected by Western blotting.

For co-immunoprecipitation, the cellular lysates (1 mg protein/ml) were precleared twice with control IgG and protein A/G agarose (Thermo Fisher), and incubated with specific Abs at 4°C for 1 h, followed by protein A/G sepharose beads overnight at 4°C. For parallel precipitations with Abs directed to different antigens, equal volumes of the same lysate were used for input. The beads were washed with RIPA buffer (New England Biolabs, Frankfurt, Germany), resuspended in electrophoresis sample buffer, and heated at 50°C for 30 min.

### Western Blotting

Cells or tissues were lysed in M-PER or T-PER lysis buffer containing Halt™ protease inhibitor cocktail (Thermo Fisher). Western blotting was performed as described (24), using the fluorochrome-labeled secondary Abs goat-anti-rabbit IRDye 680RD and goat-anti-mouse IRDye 800RD IgG (H + L) (Licor, Bad Homburg, Germany).

### Dual Luciferase Reporter Assay

In the Dual Luciferase Reporter Assay (Promega GmbH, Mannheim, Germany), a β-catenin-activated reporter (pTOPFLASH), and an unresponsive reporter (pFOPFLASH; kindly provided by B. Vogelstein, Johns Hopkins University, Baltimore, MD, USA) were used to measure the effect of CD97 on β-catenin-dependent nuclear transcriptional activity (26). 5 × 10^4^/well HEK293 cells were seeded in a 48-well plate and transiently transfected with 0.2 µg pTOPFLASH or pFOPFLASH, 0.1 µg β-catenin expression construct, and varying amounts of CD97(EGF125) mock or CD97 (125) pcDNA3.1 after 24 h (26).

### Statistics

All statistical computations (Wilcoxon–Mann–Whitney and Fisher’s-exact tests, Spearman rank order correlation) were performed using SPSS version 24.0 (SPSS, Chicago, IL, USA). *p*-Values less than 5% were considered as significant.

## Results

### CD97 Is a Potential Interaction Partner of Junctional Proteins

**Figure 1:**
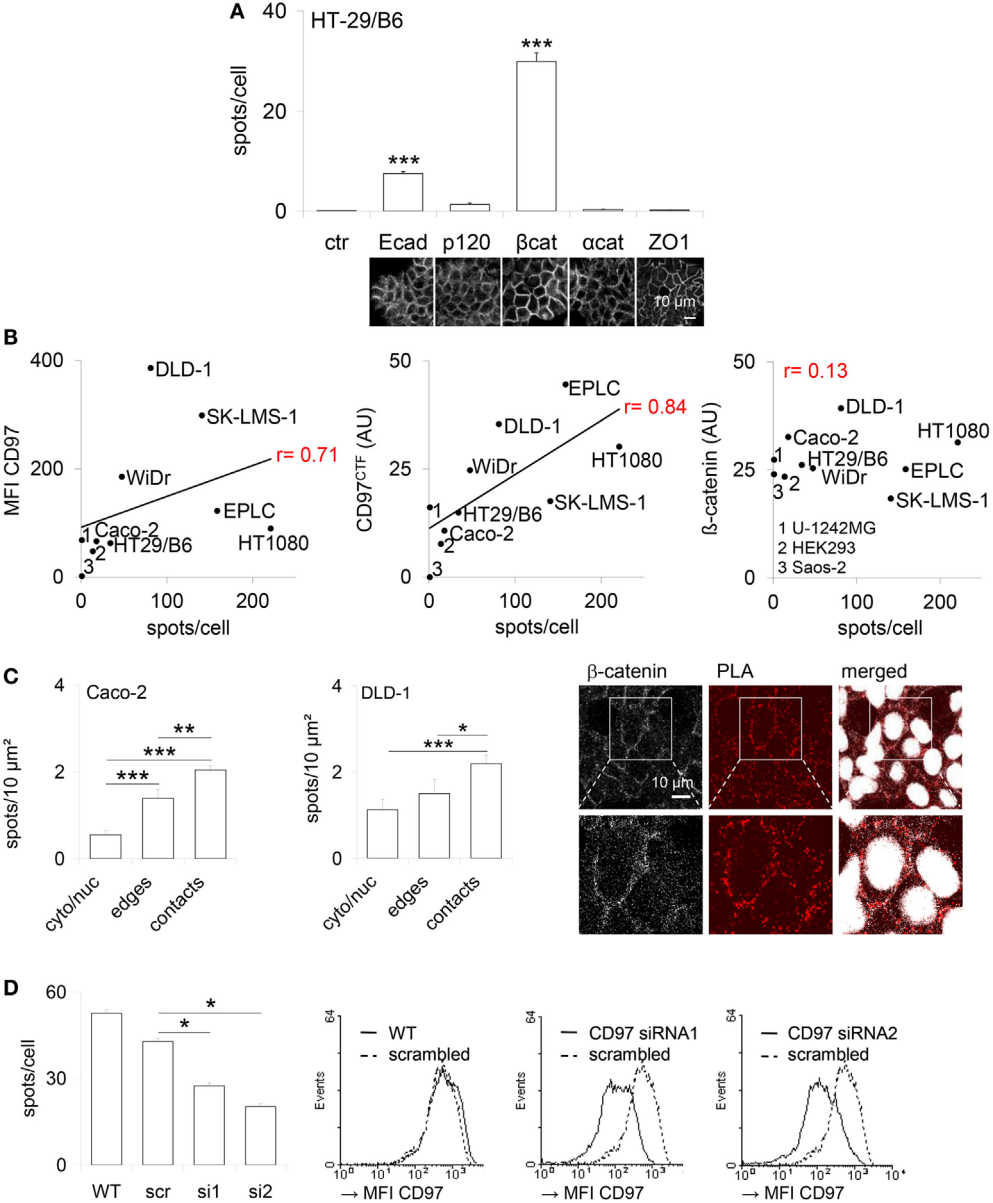
CD97 colocalizes with proteins located in adherens junctions. **(A)** Co-localization of CD97 and the adherens junction proteins E-cadherin (Ecad), p120-catenin (p120), β-catenin (βcat), and α-catenin (αcat) in HT-29/B6 cells, determined by proximity ligation assay (PLA). The number of PLA interaction spots/cell was counted. The tight junction protein ZO-1 was used as a control to estimate the background signal (mean ± SEM, *n* = 50 cells, ***p < 0.001). At the bottom, representative photographs of the immunostained cell layers are shown. **(B)** The CD97 expression level, determined by flow cytometry (mean fluorescence intensity, MFI; left) and western blotting (arbitrary units, AU; middle), and the number of CD97:β-catenin PLA interaction spots correlated well in various human tumor cell lines. Because of its uniform high expression this correlation was unverifiable for β-catenin (right). **(C)** The number of CD97:β-catenin PLA interaction spots was quantified in subcellular compartments of Caco-2 and DLD1 cells after additional staining of the bound β-catenin antibody (Ab) with a fluorochrome-labeled secondary Ab and nuclear labeling. Cell contacts showed the highest number of interaction spots (mean ± SEM, *n* = 15 cells, *p < 0.05, **p < 0.01, ***p < 0.001). Representative photographs of DLD1 cells, taken by laser scanning microscopy, are shown to the right. Interaction spots were enriched in cell contacts. **(D)** The number of CD97:β-catenin PLA interaction spots decreased in two CD97 siRNA DLD1 clones (s1, s2) compared to a scrambled (src) clone and wild-type (WT) cells (left; mean ± SEM, *n* = 4 experiments, *n* = 50 cells, *p < 0.05). Histograms (right panel) show flow-cytometric analysis of CD97 expression (MFI) of the analyzed cells and clones.

To detect CD97 interaction with junctional proteins, a PLA was performed in human colorectal HT-29/B6 (Figure 1A). The number of interaction spots was increased for CD97 with β-catenin and Ecad, but not with p120 and α-catenin. In various human cell lines the number of CD97:β-catenin interaction spots correlated well to the CD97 level quantified by flow cytometry (*p* = 0.022) and western blotting (*p* = 0.002), but not to the level of β-catenin which was always high (Figure 1B). Both proteins interacted mainly within cell contacts (Figure 1C). CD97 downregulation by siRNAs reduced the number of CD97:β-catenin interaction spots (Figure 1D).

### CD97 Co-Immunoprecipitates β-Catenin and Ecad

**Figure 2:**
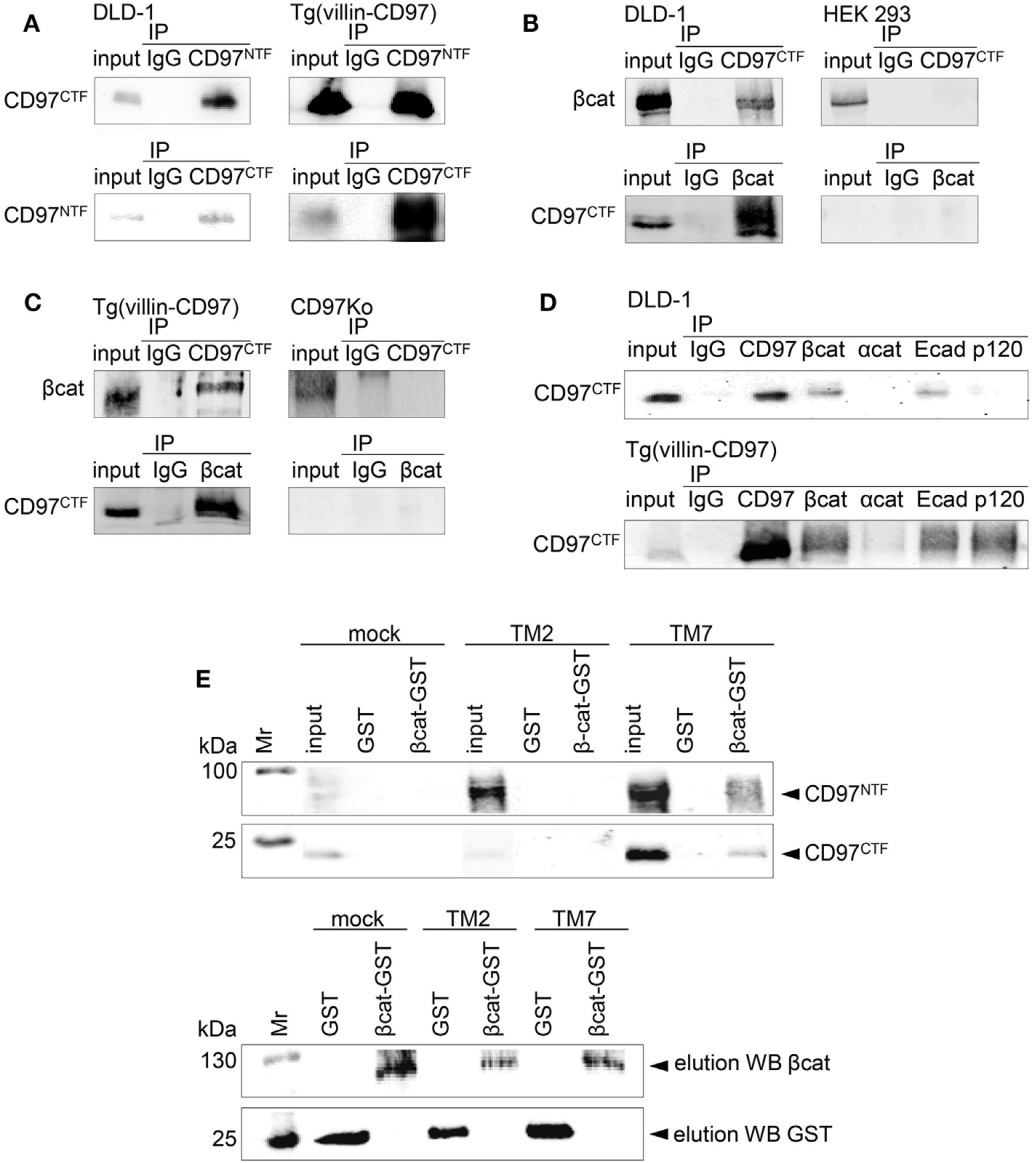
CD97 binds β-catenin. **(A)** The N-terminal (NTF) and C-terminal fragment (CTF) of CD97 were associated in DLD1 cells (left panel) and in the colon of Tg(villin-CD97) mice (right panel). Input control and immunoprecipitates (IP), obtained either with IgG or with CD97 NTF- and CTF-specific antibodies (Abs), were analyzed with a CD97 Ab detecting the other fragment, as indicated. **(B,C)** CD97 and β-catenin co-immunoprecipitated in CD97-positive DLD1 cells [**(B)**, left panel] and in colonic lysates of Tg(villin-CD97) mice [**(C)**, left panel]. HEK293 cells, only slightly positive for CD97 [**(B)**, right panel], and CD97 knock-out (Ko) mice [**(C)**, right panel] served as negative controls. **(D)** Next to β-catenin, CD97 co-immunoprecipitated with E-cadherin (Ecad) in DLD1 cells (upper picture) and with Ecad and p120-catenin in Tg(villin-CD97) mice (lower picture). **(E)** upper panel: beads loaded with glutathione S-transferase (GST) or β-catenin-GST were incubated with lysates of HT1080 CD97(EGF125), CD97(EGF125/TM2) cells with a C-terminal truncated CD97 (15), and mock cells. Bound proteins were eluted and CD97 was detected with NTF or CTF CD97-specific Abs by western blotting. Pulldown could be demonstrated only with β-catenin-GST and lysates of HT1080 cells expressing full-length (TM7), but not expressing C-terminal truncated (TM2) CD97. In the lower panel the respective elution controls for β-catenin and GST are shown.

Next, we verified biochemical binding of endogenous CD97 with β-catenin and Ecad by co-immunoprecipitations. CD97^NTF^ Ab immunoprecipitated the CD97 CTF, and *vice versa*, from DLD1 cells and the colon of Tg(villin-CD97) mice, confirming the association of both CD97 fragments in human and mouse (Figure 2A). In DLD1 cells and colonic lysates the β-catenin Ab co-immunoprecipitated the CD97 CTF, and *vice versa* (Figures 2B,C). HEK293 cells expressing sparsely CD97 and colon tissue of a *Cd97* Ko mouse served as controls. In accordance to the PLA results, CD97 co-immunoprecipitated Ecad in DLD1 cells and the colon of Tg(villin-CD97) mice (Figure 2D). In mice CD97 bound also to p120-catenin.

In pulldown experiments, we confirmed the co-immunoprecipitation results. GST-tagged β-catenin or GST, coupled to gluthatione-agarose beads, was incubated with lysates of various stable HT1080 clones (Figure 2E). The CD97 NTF and CTF were detected in eluates of cells with the full-length (TM7) CD97(EGF125), whereas lysates of cells expressing mock cDNA or a C-terminal truncated CD97(EGF125/TM2) showed no enrichments. Thus, CD97 interacted via its TM7 region or the intracellular tail with β-catenin.

### CD97 and β-Catenin Co-Localize in Various Subcellular Compartments

**Figure 3:**
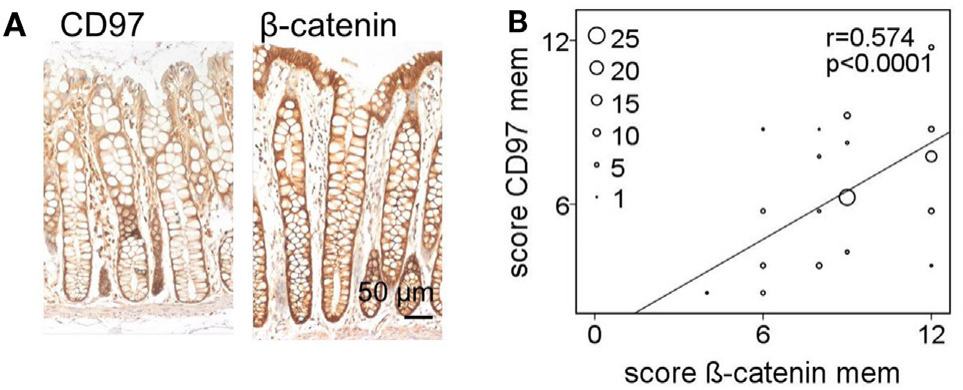
In normal colon membranous CD97 and β-catenin correlate strongly. **(A)** CD97 but not β-catenin showed an expression gradient along the crypt axis in normal colon. **(B)** Correlation of staining scores for membranous (mem) CD97 and β-catenin in normal colon. The circle size represents the number of patients (1: 1–4, 5: 5–9, 10: 10–14, 15: 15–19, 20: 20–24; 25: >25 patients).

To quantify and correlate the subcellular localizations and levels of CD97 and β-catenin *in situ*, immunostained sections of colorectal specimens were examined in detail. In normal tissue, both proteins were present in lateral enterocytic contacts (Figure 3A). CD97 showed a gradient in expression along the crypt axis in 100 out of 111 (100/111) normal tissues, i.e., CD97 was more abundant in bottom as compared to luminal crypt cells. The staining scores of CD97 and β-catenin at membranes correlated well (*p* < 0.0001), i.e., the higher CD97 was, the higher β-catenin was (Figure 3B; Table 1). Both proteins were only slightly present in the cytoplasm of normal enterocytes.

**Table 1:** Correlation of the staining score of CD97 and β-catenin in various cellular compartments in colorectal carcinomas and the corresponding normal tissue (*n* = 111 patients).

	Correlation	*r*	*p*-Value
Normal tissue	CD97 mem to β-cat mem	0.574	<0.0001
	CD97 mem to β-cat cyto	0.204	0.032
	CD97 cyto to β-cat mem	0.189	0.047
	CD97 cyto to β-cat cyto	−0.040	0.676

Colorectal cancer	CD97 mem to β-cat mem	0.154	0.106
	CD97 mem to β-cat cyto	0.206	0.030
	CD97 mem to β-cat nuc	0.113	0.236
	CD97 cyto to β-cat mem	0.070	0.466
	CD97 cyto to β-cat cyto	0.481	<0.0001
	CD97 cyto to β-cat nuc	0.146	0.127

*mem, membranous; cyto, cytoplasmic; nuc, nuclear*.

**Figure 4:**
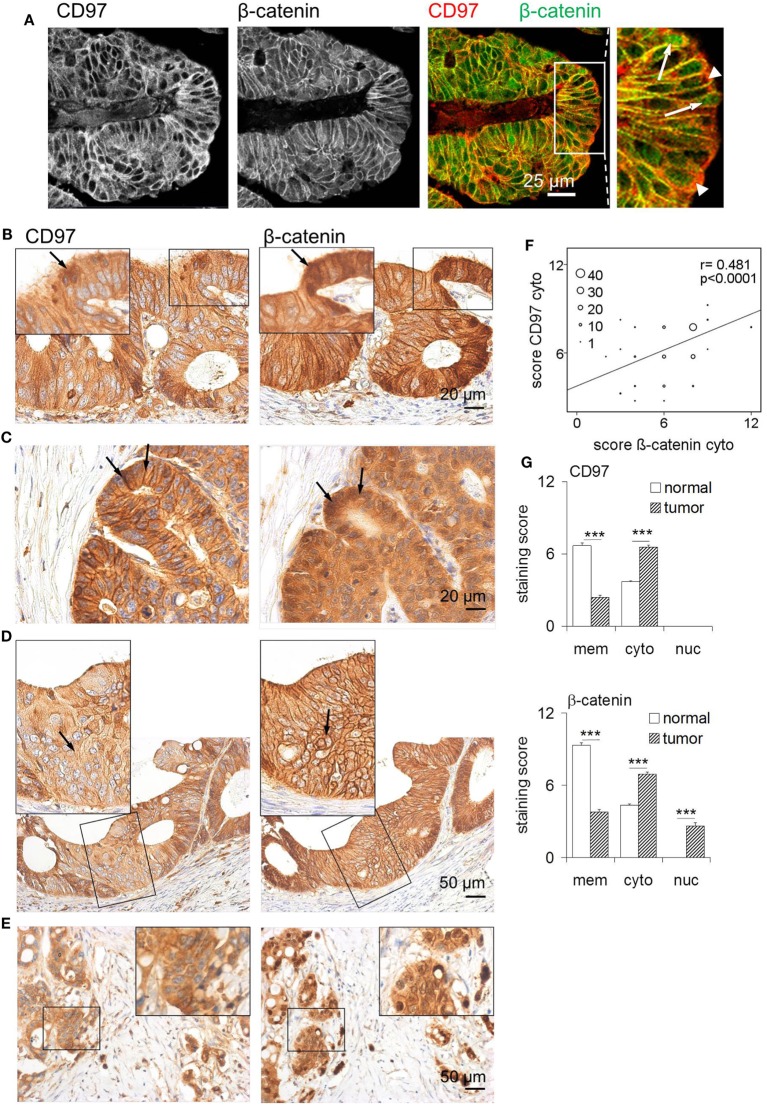
Altered localization of CD97 and β-catenin in colorectal carcinomas. **(A)** CD97 and β-catenin co-localized in lateral cell contacts of colorectal cancer cells, as seen in double-immunofluorescence stained sections. The right panel shows an overlay for CD97 and β-catenin staining. In some tumor cells, CD97 was localized in the cytoplasm (arrow heads in the larger magnification), whereas β-catenin partly appeared in the nuclei (arrows). **(B)** In parts of this tumor, CD97 was strongly present in the cytoplasm (left, arrow), whereas β-catenin occurred in the nuclei (right, arrow). **(C)** This tumor showed a strong CD97 expression mainly at the membrane, but also in the cytoplasm (left, arrows), whereas β-catenin was much more strongly expressed in the cytoplasm and nuclei (right, arrows). **(D)** In this part of a tumor, CD97 appeared more in the cytoplasm (left, arrow), whereas β-catenin was still present at the membrane (right, arrow). **(E)** In budding tumor cells at the invasion front, cytoplasmic localization of CD97 (left) and nuclear β-catenin (right) was seen. **(F)** Correlation of staining scores for cytoplasmic (cyto) CD97 and β-catenin in colorectal carcinomas (*n* = 111). The circle size represents the number of patients (1: 1–9, 10: 10–19, 20: 20–29, 30: 30–39, 40: >40 patients). **(G)** Staining scores for CD97 (upper panel) and β-catenin (lower panel) in various cellular compartments (*n* = 111 colorectal carcinomas, mean ± SEM, ***p < 0.001). In normal colon, both molecules showed highest expression at the membrane and only moderate expression in the cytoplasm. Malignant transformation caused increased cytoplasmic CD97 and cytoplasmic/nuclear β-catenin localization.

Colorectal tumors showed heterogeneity concerning the localization of CD97 and β-catenin. Typical staining patterns are shown in Figure 4 and correlations are summarized in Table 1. Double CD97 and β-catenin immunostained sections with fluorochrome-labeled secondary Abs (Figure 4A) revealed that both proteins were present at the membrane and in the cytoplasm. Only β-catenin also appeared in the nuclei.

Thus, to study tumor-associated changes in the localization of CD97 and β-catenin in detail, we quantified both proteins in subcellular compartments in serial sections of 111 colorectal carcinomas and their corresponding normal tissues. All tumors expressed CD97 at the membrane and in the cytoplasm, but never in the nuclei. Beside parts with strong membranous CD97 (Figures 4B,C), nearly all tumors (103/111) contained parts where CD97 was only weakly present at the cell membrane (Figures 4D,E), resulting in a lower staining score for membranous CD97 in malignant as compared to normal tissue. Inversely, in 92/111 specimens, the level of cytoplasmic CD97 expression was higher in malignant compared to normal enterocytes. CD97 cytoplasmic location correlated with tumor budding (*p* = 0.041) as seen in Figure 4E. As for CD97, in most specimens (102/111), the staining score for β-catenin at the membrane was lower in malignant compared to normal enterocytes, whereas in 87/111 specimens, cytoplasmic β-catenin was higher in the tumor. Nuclear accumulation of β-catenin in tumor cells was evident in 60% of the specimens (Figures 4A,C,E). Tumor budding correlated to low membranous β-catenin (*p* = 0.010) and to its nuclear presence (*p* = 0.020) in tumor cells.

Interestingly, CD97 and β-catenin decreased simultaneously from the membrane of tumor cells (*p* = 0.017). The presence of CD97 and β-catenin within the cytoplasm of tumor cells correlated strongly (*p* < 0.0001; Figure 4F; Table 1). Carcinomas with a simultaneous decrease of CD97 and β-catenin from the membrane showed increased tumor budding (*p* = 0.045). Cytoplasmic CD97 and nuclear β-catenin did not correlate. In the case of simultaneous appearance of CD97 in the cytoplasm and β-catenin in the nucleus, seen in half of the tumors, an increase in tumor budding was evident (*p* = 0.013). Figure 4G summarizes the localizations of both proteins in subcellular compartments of normal enterocytes and tumor cells. None of the parameters correlated to tumor stage.

### The Interaction of CD97 and β-Catenin Is Lost During Malignant Transformation

**Figure 5:**
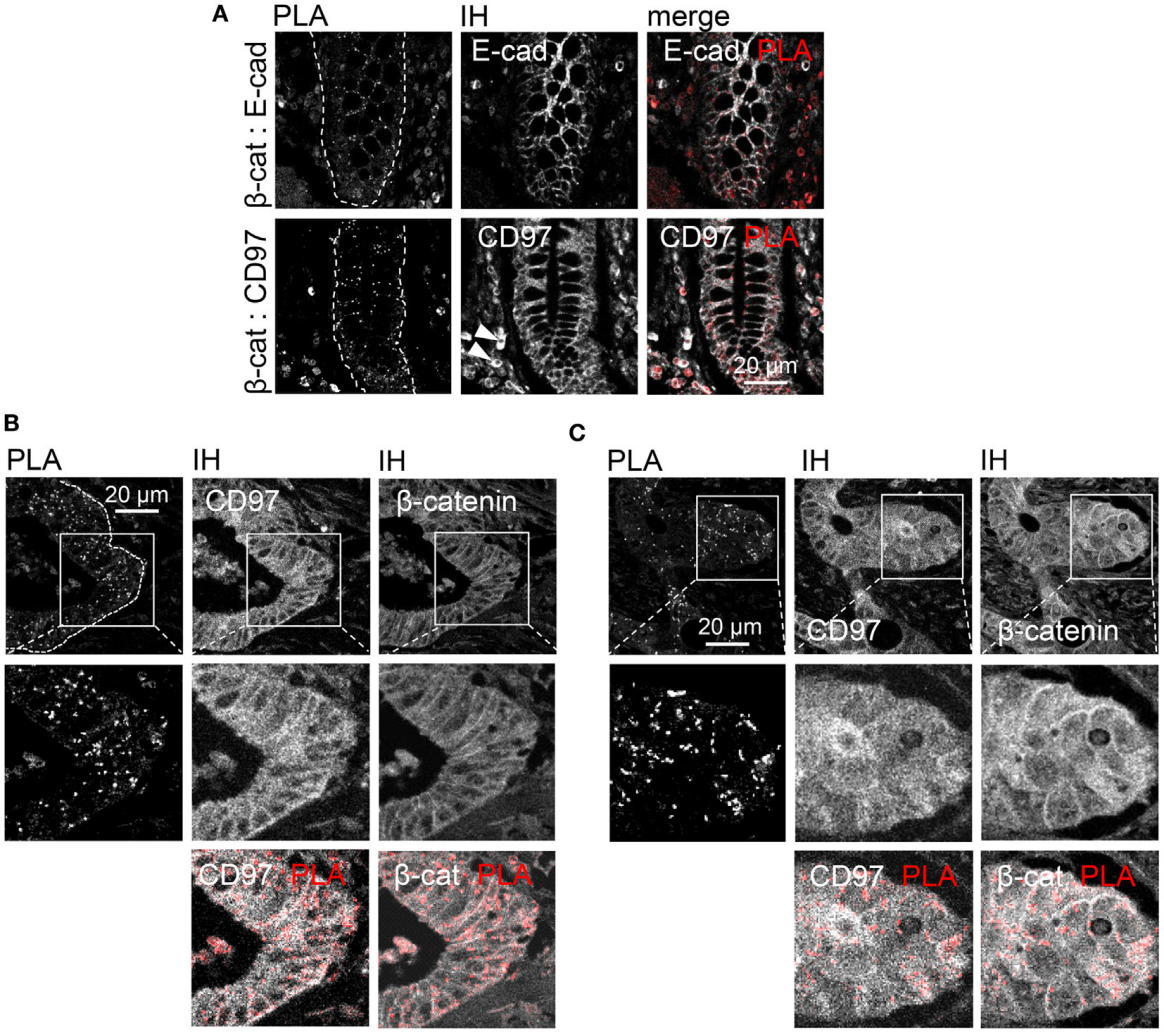
Restriction of CD97:β-catenin interaction to cellular junctions in normal colon and colorectal carcinomas. **(A)** Normal colon. Upper panel: E-cadherin:β-catenin interaction (positive control) was detected in lateral enterocytic cell contacts. Lower panel: CD97 was present in lateral and basolateral contacts of enterocytes and in leukocytes (arrow heads). CD97:β-catenin interaction was restricted to lateral junctions. **(B)** In this part of a colorectal carcinoma, mainly membranous CD97 and β-catenin expression was evident. CD97:β-catenin proximity ligation assay (PLA) interaction spots located only at the junctions. **(C)** Although CD97 and β-catenin were both expressed at high levels in the cytoplasm of this tumor, the interaction was restricted to the lateral cell contacts as seen in the PLA/immunohistology merged picture. Interaction spots are arranged as pearls at a string in the lateral contacts of tumor cells.

To verify in which cellular compartment CD97 and β-catenin interact *in situ*, a PLA was performed in tissue sections. In normal colon, interaction of CD97 with β-catenin was restricted to lateral enterocytic contacts as it was for Ecad and β-catenin (Figure 5A). In colorectal cancer, interaction spots also appeared predominantly in cell contacts, only sparsely in the cytoplasm, and never in the nuclei of tumor cells (Figure 5B). This was also evident in those tumors, where CD97 and β-catenin were mainly located in the cytoplasm (Figure 5C). Thus, the interaction of both proteins was almost always restricted to lateral junctions irrespective of their main subcellular localization. The interaction of β-catenin with an intracellular protein, adenomatous polyposis coli, is shown as a positive control in Figure S1 in Supplementary Material.

**Figure 6:**
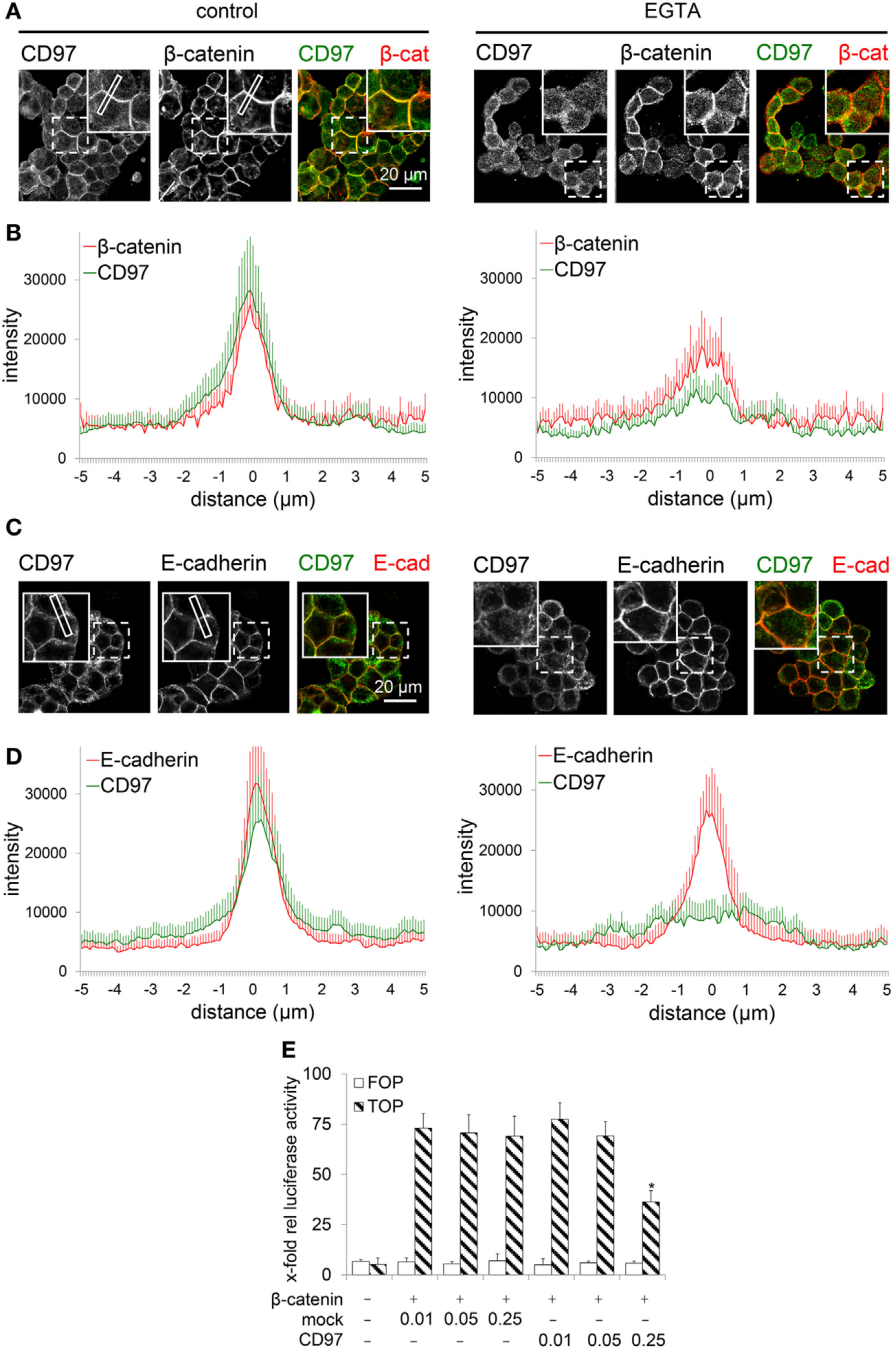
CD97 does not regulate nuclear β-catenin function. **(A,C)** In WiDr cells overexpressing a CD97-EGFP fusion protein, treatment with 3.2 mM EGTA over 20 min partly translocated CD97-EGFP into the cell, whereas β-catenin **(A)** partly and especially E-cadherin (Ecad) **(C)** still remained in the cell contacts as seen in the inserts of the pictures. As an example, a plot profile is indicated where the distribution of the various proteins has been quantified. **(B,D)**. The distribution of CD97 and β-catenin **(B)** as well as CD97 and Ecad **(D)** was analyzed in microscopic images. The staining intensity was quantified in plot profiles of 10 µm across the junctions using Fiji (*n* = 10 junctions, mean ± SEM; 0 = junctional midpoint). **(E)** CD97(EGF125) did not change β-catenin-dependent TCF-mediated transcriptional activity in a dual luciferase reporter assay with HEK293 cells (pTOPFLASH, TOP; pFOPFLASH, FOP). At high CD97 concentration (micrograms/well) even an inhibitory effect was seen (mean ± SEM, *n* = 4, *p < 0.05 mock to CD97).

To verify whether CD97 translocates into the cytoplasm after disrupting cell contacts, WiDr CD97(125)-EGFP cells were treated with EGTA. In untreated cells CD97 co-localized well with β-catenin and Ecad, respectively (Figures 6A–D). 20 min after EGTA treatment, CD97 already appeared inside the cells, whereas especially Ecad still remained at the membrane. Addition of Ca^2+^ and fetal calf serum to the medium reversed the CD97 delocalization effect (data not shown).

Finally, we examined whether CD97 alters intracellular β-catenin function. Nuclear β-catenin, bound to TCF/LEF, activates genes driving colorectal carcinogenesis (6). We confirmed in HEK293 cells with an intact Wnt-pathway, that β-catenin stimulated TCF-mediated transcriptional activity using pTOPFLASH, but not pFOPFLASH. Co-transfection of CD97 did not alter the activity in these cells (Figure 6E). At high CD97 concentration, even an inhibitory CD97 effect was seen. In SW480 cells with an activated Wnt-signal pathway, transfection of β-catenin was unnecessary. As in HEK293 cells, CD97 transfection had no effect. At high concentrations of CD97, activity was slightly inhibited (data not shown). Thus, CD97 did not regulate β-catenin-dependent TCF-mediated transcriptional activity. In summary, the interaction of CD97 with β-catenin is lost during carcinogenesis and both molecules follow different functional paths inside tumor cells.

## Discussion

Here, we identified β-catenin as a CD97 intracellular interaction partner by using PLA, co-immunoprecipitations and GST-pulldown experiments. The interaction of both proteins was restricted to cellular junctions. Although they disappeared simultaneously from the membrane and accumulated in the cytoplasm of colorectal tumor cells, CD97:β-catenin interaction spots were only localized to the membrane, even in carcinomas with high cytoplasmic and low membranous CD97 and β-catenin.

**Figure 7:**
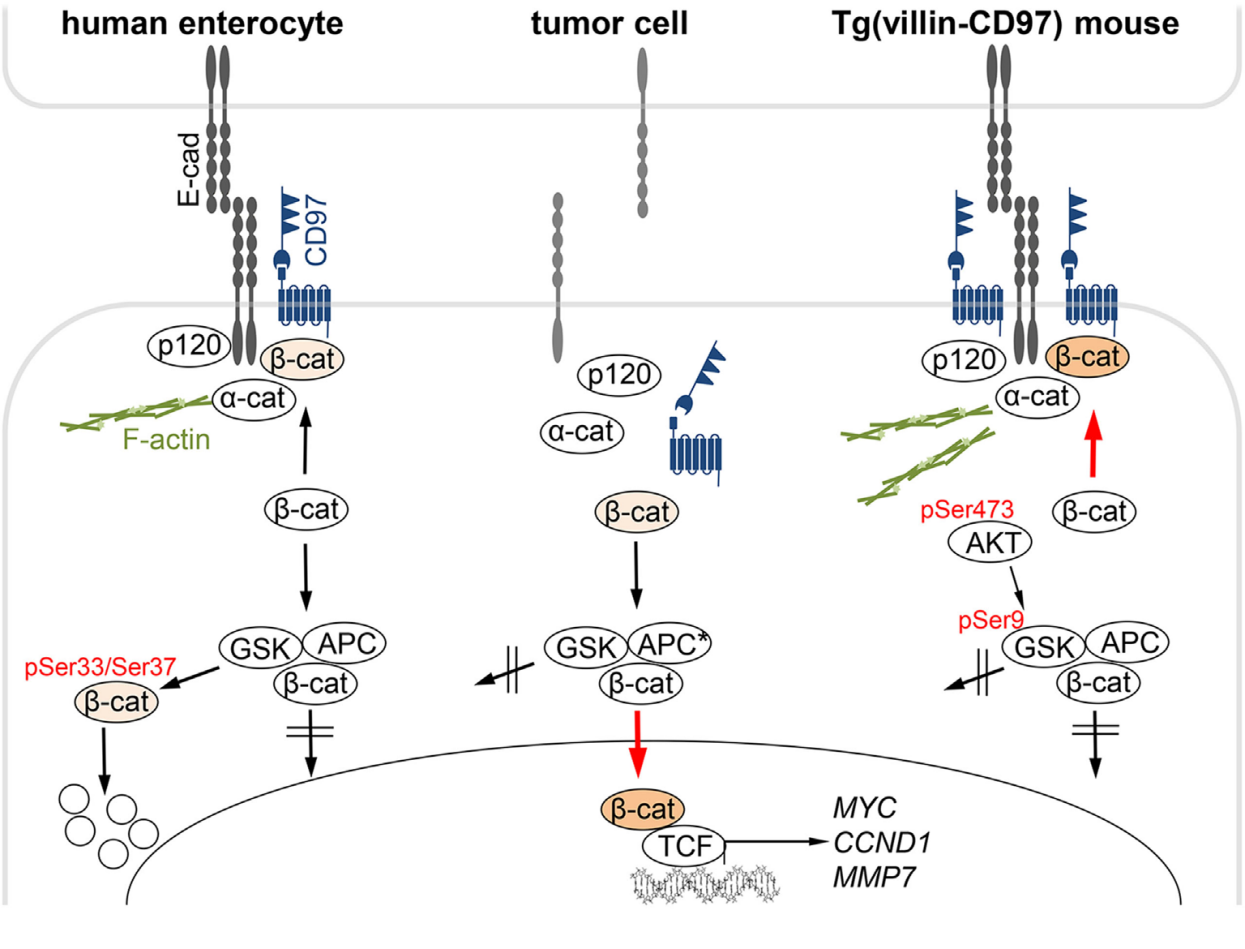
CD97:β-catenin interaction in normal and malignant intestinal epithelial cells. Left: in normal human enterocytes, CD97 is part of E-cadherin (Ecad)-based adherens junctions. CD97 physically interacts with Ecad, β-catenin, and likely, only in mice, p120-catenin, but not with a-catenin that links adherens junctions to the F-actin cortex. Cytoplasmic β-catenin is phosphorylated at Ser33/Ser37, which initiates its ubiquitination and degradation in the proteasome. Middle: In colorectal tumor cells, Ecad disappeared, and adherens junctions were dissolved. CD97 localized in the cytoplasm. In parallel, β-catenin appeared in the cytoplasm and in the nucleus, where it binds to TCF transcription factors, thus driving colorectal carcinogenesis by activating genes, such as *CCND1, MYC*, or *MMP7*. CD97 did not regulate nuclear β-catenin-dependent transcriptional activity. Right: In Tg(villin-CD97) mice, CD97 regulates the localization and stability of β-catenin in enterocytes (20). CD97 expression correlated with the amount of non-phosphorylated, stable β-catenin. AKT/GSK-3β signaling is involved in the stabilization of β-catenin through CD97. Activated AKT (pSer473) phosphorylates GSK-3β at Ser9, thereby inhibiting the ability of GSK-3β to phosphorylate β-catenin (27). In Tg(villin-CD97) mice the cytoplasmic sites of the contacts were especially condensed and actin microfilaments were pronounced. Examined phosphorylation sites (20) are indicated in red.

The results are summarized in Figure 7 that also considers our previous data on CD97 in the intestinal epithelium of transgenic mice (20). Here, CD97 stabilizes membrane-bound non-phosphorylated β-catenin, probably resulting in a strengthening of adherens junctions, which ultimately attenuates experimental colitis. In the various transgenic mouse strains which had integrated different copy numbers of *Cd97* cDNA, the CD97 level correlated well to its colitis-attenuating effect (20). Electron microscopic images feature stronger condensed adherens junctions and involved cytoskeletal filaments in Tg(villin-CD97) compared to wild-type (WT) and especially CD97-deficient mice, suggesting that CD97 modulates not only these contacts but also their cytoskeletal linkage.

To sum up, in normal epithelial cells CD97 obviously stabilized adherens junctions which is likely mediated through its interaction in *cis* with membrane-bound non-phosphorylated β-catenin and with the cytoskeleton. Interestingly, non-phosphorylated β-catenin is, as CD97 shown here, more enriched in the membrane of bottom-near compared to luminal crypt cells (28).

The highest CD97 level is evident in immune cells in which CD97 regulates mainly adhesion and trafficking [reviewed in Ref. (29)]. Whether leukocytic Ecad/catenins also interact with leukocytic CD97 has not been addressed specifically here. It is unlikely that the epithelial Ecad/catenin complex binds leukocytic CD97 in *trans* during its heterophilic adhesion with immune cells modulating their transepithelial passage [reviewed in Ref. (30)] because the interaction of CD97 with β-catenin proceeds inside cells.

The suggested junction-regulating function of CD97 in normal epithelial cells contrasts to its role in malignancies. CD97 correlates to the serum-induced migratory and invasive capacity of colorectal tumor cell lines (13). LPA has been identified as the serum component, which promotes migration and vascular extravasation of thyroid and prostate cancer through attenuating LPA-receptor signaling via Gα12/13 and RhoA by interaction with CD97 (16, 18). In the present study, the simultaneous appearance of CD97 in the cytoplasm and β-catenin in nuclei of tumor cells correlated to the appearance of tumor buds, which are thought to represent the morphological correlate of invasive cancer cells having undergone epithelial–mesenchymal transition, an important step in the progression of epithelial cancers. Tumor buds possess an anti-apoptotic behavior (31). In line herewith, CD97 inhibits apoptosis of tumor cells (32). High CD97 expression in tumor buds at the invasive front implicated that CD97 might be a direct β-catenin/TCF target gene in the deregulated canonical Wnt-pathway of colorectal cancers. However, CD97 mRNA expression levels were not affected by TCFs, as determined in inducible dominant-negative TCF colorectal tumor cell lines, and co-expression of WT or S33A-mutated β-catenin with TCF-4 did not enhance *CD97* promoter activity (26).

A question that remains to be answered is whether the different CD97 functions, regulating either cell contacts of normal epithelial cells or migration and invasion of tumor cells, are related. Although there is no conclusive evidence that a decrease in membranous CD97 leads to the cytoplasmic appearance of CD97, the fact that CD97 translocated into the cell after disrupting cell contacts, as shown here, makes a translocation instead of an accumulation and/or block of transport to the membrane in tumor cells more likely. After junctional disintegration, CD97 accumulated more in the cytoplasm than Ecad and β-catenin and their co-localization disappeared. Accordingly, CD97 did not stimulate β-catenin-dependent TCF-mediated transcriptional activity, showing that cytoplasmic CD97 does not regulate cytoplasmic/nuclear β-catenin. At high CD97 levels, even an inhibitory effect of CD97 was seen. In other words, in tumor cells CD97 and β-catenin go along intracellularly separate functional paths.

In summary, CD97 is a multifunctional aGPCR that regulates cell contacts, *via* β-catenin, in (normal) epithelial cells and cell migration and invasion in tumor cells. Depending on the subcellular context and related molecular interaction partners, altered functions of this aGPCR occur.

## Ethics Statement

This study was carried out in accordance with the recommendations of the Ethics Committee of the Medical Faculty of the Leipzig University (no. 028/2000 and 111/2009). All subjects gave written informed consent in accordance with the Declaration of Helsinki.

## Author Contributions

Conceptualization: GA; methodology: EW and GA; investigation: DH, ND, EW, SG, DS, and GA; writing original draft: GA and JH; funding acquisition: GA; resources: JH. All authors agree to be accountable for the content of the work.

## Conflict of Interest Statement

The authors declare that the research was conducted in the absence of any commercial or financial relationships that could be construed as a potential conflict of interest.

## Supplementary Material

Figure S1 Adenomatous polyposis coli (APC):β-catenin interaction verified by proximity ligation assay (PLA). Co-immunohistology (IH) of both, APC and β-catenin, was performed after PLA by detecting the primary antibodies (Abs) with fluorochrome-labeled secondary Abs. Merged pictures localized PLA interaction spots in the immunostained sections. **(A)** Normal colon: APC:β-catenin interaction was restricted to intestinal epithelial cells. **(B)** Colorectal carcinoma.

## Abbreviations

Ab, antibody; CTF, C-terminal fragment; GPCR, G-protein-coupled receptor; GST, glutathione S-transferase; LPA, lysophosphatidic acid; NTF, N-terminal fragment; PLA, proximity ligation assay; TM7, seven-span transmembrane; TM2, two-span transmembrane; Tg, transgenic.
