# Supplementary figures and images for: The Interaction of CD97/ADGRE5 With β-Catenin in Adherens Junctions Is Lost During Colorectal Carcinogenesis
Source: Front Oncol. 2018 May 25;8:182. doi: 10.3389/fonc.2018.00182 (PMC5980956; doi:10.3389/fonc.2018.00182)

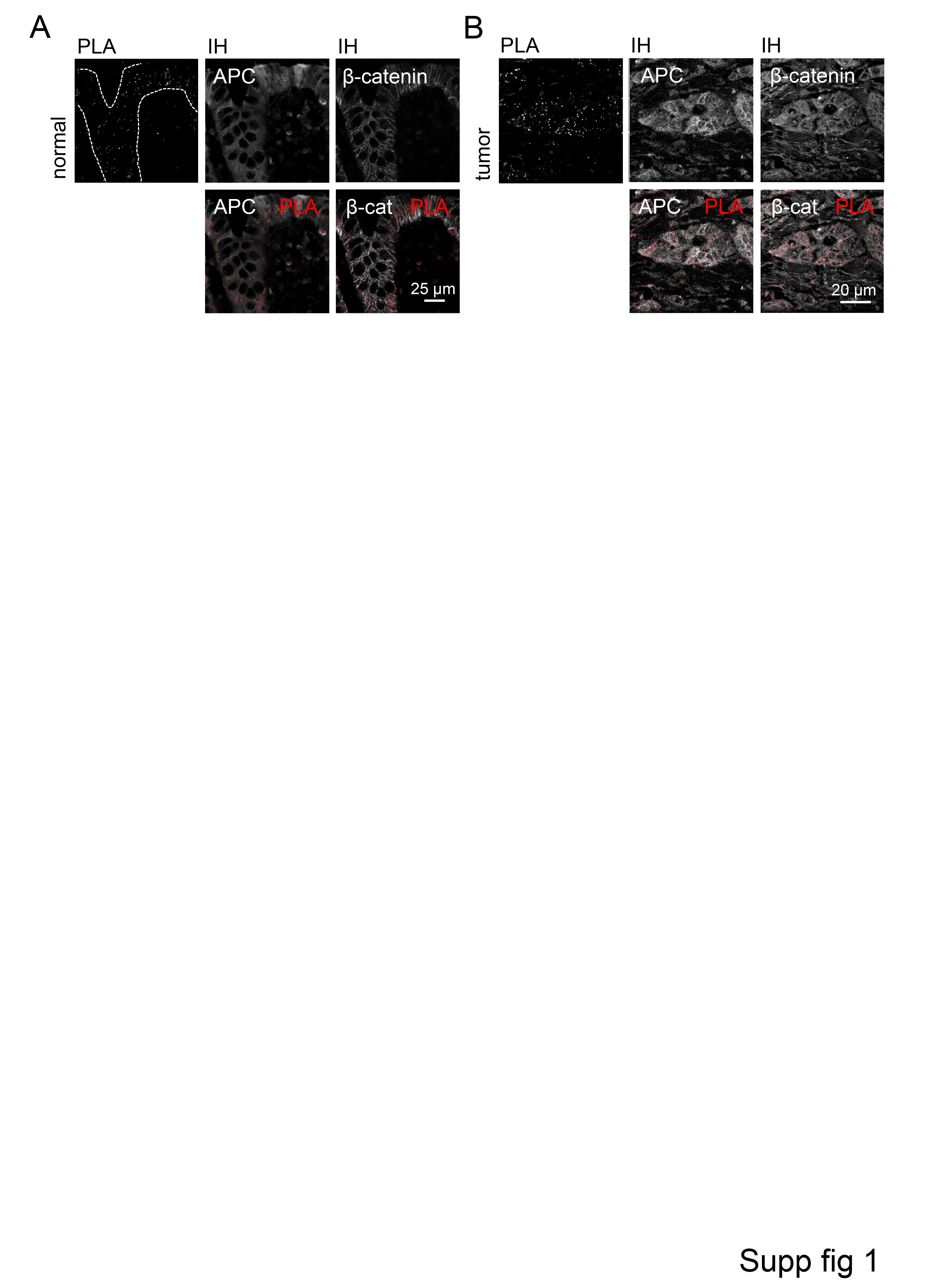

Supplement: Figure S1 — Adenomatous polyposis coli (APC):β-catenin interaction verified by proximity ligation assay (PLA). Co-immunohistology (IH) of both, APC and β-catenin, was performed after PLA by detecting the primary antibodies (Abs) with fluorochrome-labeled secondary Abs. Merged pictures localized PLA interaction spots in the immunostained sections. (A) Normal colon: APC:β-catenin interaction was restricted to intestinal epithelial cells. (B) Colorectal carcinoma. [file Image_1.JPEG]
